# Tension-type headache and low back pain reconsidered

**DOI:** 10.3389/fneur.2022.912348

**Published:** 2022-07-29

**Authors:** Jens Astrup, Finn Gyntelberg

**Affiliations:** Department of Neurology, Bispebjerg Hospital, University of Copenhagen, Copenhagen, Denmark

**Keywords:** tension-type headache, low back pain, neck pain, myofascial pain, whiplash, spinal dyssynergia, EMG

## Abstract

The natural history and clinical course of tension-type headache and non-specific low back pain are reconsidered. By closer examination, these two conditions appear to share several specific clinical features. Both are muscular pain conditions along the spine, they have a preponderance in women, they may occur spontaneously or follow a trivial traumatic incident, and they both have a high risk of chronicity. The affected muscles are tender with tender points. EMG indicates diffuse hyperactivity and abnormal activation pattern, and motor control of the affected muscles and adjacent muscle groups is discoordinated. These shared features suggest analogous pathophysiology involving the neuromotor control of affected and adjacent muscle groups in the cervical and lumbar regions, respectively. As recently suggested for the whiplash disease, we suggest the term spinal dyssynergia for this specific pattern of pathology. This suggestion provides a new perspective for the understanding of these diseases by placing their cause within the central nervous system and not in the spine or spinal musculature. This perspective warrants further clinical, neurophysiological, and neuropharmacological studies of this ‘family’ of common yet poorly understood clinical muscular pain conditions along the spine.

## Introduction

This overview reconsiders the natural history and clinical features of muscular pain conditions, tension-type headache, and non-specific low back pain to reveal possible similarities suggestive of a shared pathology. These two common chronic muscular pain conditions along the spine have certainly been extensively studied in many contexts, but they remain elusive in terms of specific peripheral pathophysiology within the spine or spinal musculature and in terms of a possible specific causative therapy. Alternatively, central pathophysiology located within the central nervous system may be considered a possible coherent cause of the essential clinical features shared by these conditions.

### Tension-type headache

Tension-type headache is a chronic muscular pain condition in the cervical-cranial spine region (1). It occurs spontaneously but may also occur following a minor head injury (2). The condition shows preponderance in women and a tendency to become chronic. In the present context of a possible central cause of this condition, it is of particular interest that tension-type headache patients display discoordination of head movements and discoordination of adjacent muscle groups. The discoordination of head movements was first observed by Woodhouse and Vasseljen (3) and Kristjansson and Oddsdottir (4) using ‘the fly method’ and recently confirmed by Astrup et al. (5) using the laser tracking technique. This evidence indicates impaired neuromotor control by the central nervous system. It seems reasonable to consider this discoordination of head movements to be a functional expression of the EMG abnormalities in the neck muscles observed in these patients. These are diffuse hyperactivity as indicated by increased mean root square index, which correlates to muscle tenderness, and an abnormal activation pattern, such as co-activation of accessory muscles (6–8). Even co-activation of antagonist's muscles as in dystonia may be observed (8). Some of these authors state that their observations are signs of a “spinal hyperexcitability” and neural “reorganization of motor control strategy.” Furthermore, adjacent muscle groups, such as those controlling the upper limb (9, 10) and the eyes (11), are discoordinated in patients with tension-type headaches. Similar discoordination of movements and similar EMG changes have been observed in patients with whiplash (12–16). These impairments of movement patterns in neck pain have been reviewed by Hesby et al. (17).

### Non-specific low back pain

Non-specific low back pain is a chronic muscular pain condition in the lumbar spine region. It may occur spontaneously or may follow a minor back-related incident. It may also occur in low-velocity rear-end car collisions either solely or in conjunction with whiplash (18). The condition shows a preponderance in women and a tendency to become chronic (19). EMG has been less studied but like in chronic neck pain conditions, a pattern of diffuse hyperactivity and abnormal activation has been observed (20, 21). Furthermore, patients with acute and chronic non-specific low back pain display postural imbalance measured as sway when standing on a balance pad (22–25). This imbalance may in this context be considered as the functional expression of a neuromotor discoordination similar to the discoordination of head movements observed in patients with tension-type headache. Also, similar to tension-type headache, adjacent muscle groups demonstrate an altered activation pattern, which in patients with low back pain is observed in the trunk musculature (26).

### Indications of analogous pathophysiology in tension-type headache and non-specific low back pain

The evidence indicated above shows several shared analogous clinical and neurophysiological features of tension-type headache and non-specific low back pain affecting the cervical and the lumbar musculature, respectively. These are listed in Table 1.

**Table 1:** Summarizes the shared analogous features in natural history and clinical course of tension-type headache and non-specific low back pain.

**Features**	**Tension-type headache**	**Non-specific low back pain**
Occur spontaneously	Yes	Yes^**^
May occur following minor traumatic incidence	Yes ^*^	Yes^**^
Signs of initial structural injury	None	None^**^
Symptom delay^***^		Yes^**^
Risk of Chronicity	High	High
Specific therapy	No	No
Female preponderance	Yes	Yes
EMG	Increased	Increased
EMG activation pattern	Abnormal	Abnormal
Muscle tenderness and tender points	Yes	Yes
Impaired motor control of affected muscles	Yes (head movements)	Yes (postural sway)
Impaired motor control of adjacent muscle groups	Yes (eye and shoulder girdle/upper limb)	Yes (trunk)

* *Tension-type headache may occur as a complication of a minor head injury (2)*.

** *The epidemiology of non-specific low back pain has been well-studied in occupational and socio-economic contexts, but less in terms of initiation, trauma or no trauma, possible symptom delay, determinants of chronicity, and sex differences. Consequently, the indications of these parameters related to non-specific low back pain in this table are merely qualitative indicators of ‘common knowledge' since more exact measures are not or are only partly available (19)*.

*** *Symptom delay refers to the delay which often occurs between the initial traumatic back incident/overload and the appearance of low back pain. This phenomenon indicates a developing pain condition rather than a direct traumatic strain of myofascial fibers*.

The number and nature of these shared analogous clinical features provide a background for the consideration of common causative pathophysiology for these two conditions of chronic muscular pain along the spine. The EMG changes and associated impaired motor discoordination point toward a neurological dysfunction located within the central nervous system as an alternative to the peripheral spinal structures. Furthermore, other features, such as the initiation of these conditions, either spontaneously or following a low energy incident without signs of structural injury and their high rate of chronicity, make a central rather than a peripheral cause of these conditions more coherent. We consider it unlikely that a peripheral cause may induce EMG changes and discoordination of affected and adjacent muscle groups, that a peripheral cause may initiate these conditions spontaneously or following a low energy incident, and that a peripheral cause may carry a high rate of chronicity. Instead, it appears reasonable to consider this pattern of shared analogous features as signs of a central pathology as the primary event. We suggest the term *spinal dyssynergia* for this central pathology, which essentially initiates as a state of hyperactivity an abnormal activation pattern of the involved muscle groups causing muscular tenderness and may subsequently lead to a chronic pain condition. If so, this implies that the central nervous system tends to develop such a state of spinal dyssynergia either spontaneously or following a trivial low-energy physical event. Furthermore, this may happen quite often since these conditions are very common. Once this state of dyssynergia has emerged, it cannot be reversed by any known therapy, and it may even remain chronic for many years. This appears as an inappropriate mode of reaction by the central nervous system, but in analogy, to the focal dystonias, it may not be an unlikely mode of reaction. Focal dystonias show some similarities. Cervical dystonia (spasmodic torticollis), for example, is a dysfunction of the neuromotor control of the neck muscles, which may occur spontaneously or less often following an external physical event affecting the neck (27, 28). This dysfunction is focal and synergic in the sense that the overactivated neck muscles act together in turning the head in one direction. The hyperactivity in spinal dyssynergia, however, is diffuse on a regional level and dyssynergic. The focal dystonias indicate that a focal neuromotor dysfunction may occur within the central nervous system either spontaneously or following a trivial incident. Accordingly, we conclude that the central nervous system has an inappropriate ability under certain circumstances and in predisposed individuals to develop focal or regional neuromotor dysfunctions, either synergistic as in focal dystonias or dyssynergistic as in spinal dyssynergias. As a hypothesis, it may explain some of the features of the muscular pain conditions along the spine. It may coexist with the phenomenon of sensitization of the nociceptive system, which occurs as a non-specific reactivity of the central nervous system to chronic pain of any kind including tension-type headache and low back pain (29).

Further studies are warranted. Recently, we reconsidered the whiplash disease grade 2 and concluded that its natural cause and clinical features are better explained as a neurological rather than a post-traumatic condition and we suggested the term spinal cervical dyssynergia to describe the neurology of this condition (30). In a previous study, we observed that patients with whiplash disease grade 2 presented with identical clinical symptoms and signs and head movement discoordination as observed in patients with tension-type headaches (5), and we suggested that tension-type headache and whiplash disease may be regarded as the same basic disease occurring either spontaneously or initiated by a minor low energy trauma, respectively.

The present perspective suggests that non-specific low back pain, tension-type headache, and whiplash disease represent a family of muscular pain conditions along the spine caused by a state of spinal dyssynergia affecting the neuromotor control in the respective cervical and lumbar regions. This view is controversial in the sense that it suggests that these common pain conditions along the spine may share the same basic causative neuropathology, and accordingly should be considered a ‘family’ of related neurological disorders, and concurs with the highly significant clinical coexistence between tension-type headache and low back pain (31). This paradigm warrants further investigations within such a context and may open a window for specific neuropharmacological interventions directed toward dysfunctional neuromotor control.

## Data availability statement

The original contributions presented in the study are included in the article/supplementary material, further inquiries can be directed to the corresponding author.

## Author contributions

JA prepared the manuscript. FG has assisted in preparing the manuscript. All authors contributed to the article and approved the submitted version.

## Conflict of interest

The authors declare that the research was conducted in the absence of any commercial or financial relationships that could be construed as a potential conflict of interest.

## Publisher's note

All claims expressed in this article are solely those of the authors and do not necessarily represent those of their affiliated organizations, or those of the publisher, the editors and the reviewers. Any product that may be evaluated in this article, or claim that may be made by its manufacturer, is not guaranteed or endorsed by the publisher.
